# Defining clinical endpoints in limb girdle muscular dystrophy: a GRASP-LGMD study

**DOI:** 10.1186/s12883-024-03588-1

**Published:** 2024-03-15

**Authors:** Amy Doody, Lindsay Alfano, Jordi Diaz-Manera, Linda Lowes, Tahseen Mozaffar, Katherine D. Mathews, Conrad C. Weihl, Matthew Wicklund, Man Hung, Jeffrey Statland, Nicholas E. Johnson, On behalf of the GRASP-LGMD Consortium, Kathy Mathews, Doris Leung, Peter Kang, Urvi Desai, John Vissing, Carla Zingariello, Stacy Dixon

**Affiliations:** 1 Virginia Commonwealth University, Richmond, VA USA; 2 Nationwide Children’s Hospital, Columbus, OH USA; 3 Newcastle University, Newcastle, UK; 4 University of California, Irvine, CA USA; 5 University of Iowa Carver College of Medicine, Iowa City, IA USA; 6 Washington University, St. Louis, MO USA; 7 UT Health San Antonio, San Antonio, TX USA; 8 Roseman University, Salt Lake City, UT USA; 9 University of Kansas Medical Center, Kansas City, KS USA

**Keywords:** Limb girdle muscular dystrophy, Muscular dystrophy, Clinical outcome assessments, Clinical trials, Therapeutic development

## Abstract

**Background:**

The Limb Girdle Muscular Dystrophies (LGMDs) are characterized by progressive weakness of the shoulder and hip girdle muscles as a result of over 30 different genetic mutations. This study is designed to develop clinical outcome assessments across the group of disorders.

**Methods/design:**

The primary goal of this study is to evaluate the utility of a set of outcome measures on a wide range of LGMD phenotypes and ability levels to determine if it would be possible to use similar outcomes between individuals with different phenotypes. We will perform a multi-center, 12-month study of 188 LGMD patients within the established Genetic Resolution and Assessments Solving Phenotypes in LGMD (GRASP-LGMD) Research Consortium, which is comprised of 11 sites in the United States and 2 sites in Europe. Enrolled patients will be clinically affected and have mutations in CAPN3 (LGMDR1), ANO5 (LGMDR12), DYSF (LGMDR2), DNAJB6 (LGMDD1), SGCA (LGMDR3), SGCB (LGMDR4), SGCD (LGMDR6), or SGCG (LGMDR5, or FKRP-related (LGMDR9).

**Discussion:**

To the best of our knowledge, this will be the largest consortium organized to prospectively validate clinical outcome assessments (COAs) in LGMD at its completion. These assessments will help clinical trial readiness by identifying reliable, valid, and responsive outcome measures as well as providing data driven clinical trial decision making for future clinical trials on therapeutic agents for LGMD. The results of this study will permit more efficient clinical trial design. All relevant data will be made available for investigators or companies involved in LGMD therapeutic development upon conclusion of this study as applicable.

**Trial registration:**

Clinicaltrials.gov NCT03981289; Date of registration: 6/10/2019.

## Background

Limb Girdle Muscular Dystrophy (LGMD) is defined as a group of disorders with a common phenotype of progressive weakness of the shoulder and hip girdle muscles due to loss of muscle fibers. Disease progression tends to be symmetrical, but overall clinical severity and age of onset vastly differs based on genetic variant. LGMD gene mutations produce a variety of clinical phenotypes, including severe childhood-onset forms, distal and proximal myopathies, pseudo-metabolic myopathies, eosinophilic myositis, and hyperCKemia [1]. Over 30 different genetic mutations are known to cause LGMD and have either an autosomal dominant or autosomal recessive pattern of inheritance [2]. Genetic heterogeneity may also result in atypical symptom presentation (i.e., distal weakness) or cause additional symptoms such as cardiomyopathies, respiratory failure, joint contractures, and muscle pain. The large degree of clinical variability complicates not only predictions of disease progression but also clinical trial design.

Individually, each mutation is rare, however, as a collective LGMDs are one of the four most common muscular dystrophies. Despite the incidence, LGMD epidemiological studies are quite limited. A meta-analysis of studies published since 1991 provided a global incidence of 0.7/100,000 and prevalence of 3/100,000 [1]. Projected worldwide estimates would yield approximately 10,050 affected individuals within the United States. Despite limited population prevalence studies, there are many studies looking at the relative frequency of different LGMD subtypes. A study from 2006 found a US distribution of LGMD mutations: 18% DYSF (LGMDR2), 15% SGCA (LGMDR3), 15% FKRP (LGMDR9), 12% CAPN3 (LGMDR1), and 1.5% CAV3 (LGMD1C) [3]. Prior limitations to data collection on LGMD subtype preferences are in part due to the high cost of genetic sequencing. Programs by the Muscular Dystrophy Association, the Jain Foundation, and others have provided free genetic testing for individuals with LGMD in the US to combat this barrier. The 35-gene LGMD panel, performed through Emory University from June 2015 through June 2017, has provided a relative prevalence of mutations within the 4656 individuals who had genetic testing [4].

The four most common recessive LGMD mutations which might be amenable to gene replacement were in CAPN3 (LGMDR1, 17%), DYSF (LGMD2B, 16%), FKRP (LGMDR9, 9%) and ANO5 (LGMDR12, 7%). These LGMDs are prime candidates for gene therapy and were the ideal LGMD subtypes to focus on in this project [4].

The genetic heterogeneity of LGMD has been a barrier to broad natural history efforts, with prior investigations generally limited to single gene mutations. Much attention is paid to the variability within individual mutations (e.g., distal presentations), as opposed to defining the best strategy for measuring change in overall LGMD disease burden. This creates a major dilemma for LGMD rare disease research: how to balance diverse genes leading to overlapping phenotypes, versus variants in the same gene leading to divergent phenotypes. What is clear is as a group, LGMDs are chronic and progressive leading to significant lifetime morbidity and represent a large unmet clinical need.

### Drug development landscape

Previous research in inherited muscular disorders suggests that therapies directly targeting gene mutations are possible [5–7]. Currently, there are three approved treatments for spinal muscular atrophy (SMA). These are Nusinersen (Spinraza), an antisense oligonucleotide, Onasemnogene abeparvovec-xioi (Zolgensma), an SMN1 gene replacement, and Risdiplam (Evrysdi), a small molecule SMN2 modifier [8]. Multiple antisense-based therapies (exon 53, 51, and 45 skipping) are approved in the United States as therapies for Duchenne Muscular Dystrophy (DMD) [9]. One gene therapy, Delandristrogene moxeparvovec, is also approved for treatment of ambulatory DMD patients in the US [10]. Additionally, Europe has provisionally approved a drug that causes read-through of premature stop mutations in DMD [11].

Recent developments in our genetic understanding of LGMD and molecular approaches to therapy have led to proposed gene replacement therapies for at least three LGMD mutations. Phase 1 studies of AAV-delivered gene therapy for LGMD 2C and D (sarcoglycan) have demonstrated proof-of-principle for delivery in an isolated muscle and showed sarcoglycan staining in muscle biopsies post therapy [12–15]. Preclinical efforts to develop gene therapies for FKRP mutations (LGMDR9) and sarcoglycan B mutations (LGMDR4) are underway [16–22]. For the dominant LGMDs which are not amenable to gene replacement, there is evidence that small molecules can affect the pathology associated with DNAJB6 mutations (LGMDD1) [23]. With multiple gene replacement therapies currently in pre-clinical/phase 1 testing, there is a demand for natural history data to guide trials [24].

In addition to molecular based therapies, many non-specific muscular dystrophy therapies are currently in clinical trials. Such approaches may prove beneficial for LGMD. These include targeting the myostatin pathway, muscle inflammation, regeneration, or fibrosis. Recently completed early phase studies of non-specific therapies in LGMD include an anti-myostatin drug to improve muscle growth (NCT02841267), a steroid to target inflammation (NCT00527228), and a tRNA synthetase to target muscle inflammation and regeneration (NCYT02836418).

Similarly, based on pathogenesis, an approach that stabilizes or repairs the sarcolemmal membrane would also be applicable across LGMDs. Therefore, it would be important, for example, to understand if the clinical outcomes assessments sensitive for LGMDR2 progression would be sensitive in people with LGMDR1 mutations. The rapid advancement of molecular based therapeutics in inherited neuromuscular disorders, and gene-based therapies for LGMD create an urgent need to validate clinical outcome assessments (COAs) for LGMD. A more efficient approach to quickly evaluating multiple LGMD subtypes simultaneously would advance drug development for LGMD more rapidly, resulting in better patient outcomes.

### Clinical trial readiness

Validated COAs that quantify functional abilities in persons with LGMD would serve as a valuable resource for drug trial assessment. Through discussions with academic researchers and industry partners, several gaps have been identified to overcome in LGMD trial preparedness to effectively and efficiently assess the numerous candidate therapeutics projected to enter larger studies in the next 5 years. These include 1) better diagnostics; 2) refined trial strategies for inclusion or exclusion criteria; 3) biomarkers for early phase studies; 4) COAs and patient-reported outcomes for later phase studies and drug registration trials; and 5) understanding baseline variables which may predict more consistent progression over time frames typical for clinical trials (e.g., 1 year). A major barrier to therapy development for LGMD is lack of validated COAs and an inability to determine the natural history of LGMD as measured by COAs. In addition, there is limited data regarding which may be most sensitive to change over time, and which may provide more specificity for atypical phenotypic presentation.

### Prior COA exploration in LGMDs

There are many cross-sectional studies in LGMD that seek to describe the genotype–phenotype spectrum associated with individual mutations. Some strategy for grouping mutations by type is required, for example nonsense mutations, microdeletions or insertions, and missense mutations. On average, the ability for specific mutations to predict the clinical severity, age at diagnosis, or age at first wheelchair use is not strong [25–27]. Thus, disease presentation and progression across LGMD subtypes is likely heterogenous and multifactorial. Prospective, longitudinal study of available COA to evaluate validity, reliability, sensitivity to change, and meaningful change over time can enable a data-driven approach to clinical trial design. Similarly, validated outcomes can also better elucidate differences and similarities between LGMD-subtypes in terms of age at symptoms onset, clusters of presenting systems, and varying trajectories. For instance, sarcoglycanopathies typically have a younger age of onset with more prominent respiratory and cardiac morbidity than the LGMDs due to dysferlin or calpain-3 loss of function variants.

Research for LGMD is primarily focused on cross-sectional or retrospective investigation rather than longitudinal studies. Prospective longitudinal studies of individuals with CAPN3, FKRP, and DSYF mutations (LGMDR1, R9, and R2, respectively) are incomparable due to differences in inclusion criteria (e.g. age or functional status), number of participants, procedures performed, evaluator training, and/or differing follow up timing [25, 27, 28] However, these studies have been successful in demonstrating an ability to use common muscular dystrophy COAs for reliable measurement of severity of weakness or disability in a cross-sectional manner. Because it is unclear how generalizable these data are across LGMDs or the multi-site reproducibility of the results, a larger effort is vital for therapeutic development for LGMD.

Measurement tools for patterns and degree of muscle involvement have included manual muscle testing and quantitative myometry. Muscle strength as measured by manual muscle testing can illustrate patterns of weakness but has been found less sensitive to change over time when compared to quantitative muscle testing using handheld dynamometers or fixed frame systems in a large multisite cohort of patients with LGMDR2 [29]. Similarly, this study highlights the challenges of interpretations of changes in strength testing due to high variability in measurements across visits.

Magnetic resonance imaging (MRI) has been proposed in LGMD as an early biomarker to track disease progression over time frames where strength of function might not be expended to change, or within studies with a smaller cohort. The most common methods used include quantitative fat as measured by the DIXON technique, or quantitative T2. One study showed changes on MRI in LGMDR9 over 1 year despite no change in strength or function [28]. Several studies had results showing common features such as limb-girdle patterns of muscle involvement that can be useful to differentiate LGMD from other inherited muscle conditions [30]. Variability has also been evident in aspects including thigh or arm involvement [25, 27, 28]. Interestingly, MRI data has suggested that some separately defined phenotypes may represent an LGMD spectrum. For LGMDR9 (FKRP) and LGMDR2 (DSYF), use of MRI not only reinforced the limb-girdle weakness pattern of muscle involvement but also showed early and frequent involvement of more distal muscles such as the gastrocnemius [31]. MRI follow up of LGMDR9 one year later revealed anticipated evolutions in quantitative fat in the posterior thigh muscles and also demonstrated muscular changes in the gastrocnemius [28]. Moreover, water T2 has recently been suggested as a biomarker to differentiate between quick and slow progressors [32]. MRI shows potential to be important in LGMD therapeutic development; however, several limitations prohibit its widespread use at this time. Barrier to use include 1) high recurrent costs; 2) lack of common equipment and protocols; 3) reliability/variability in measurement across sites and equipment, 4) patient limitations on ability to tolerate MRI (e.g., respiratory status, contractures); 5) uncertain regulatory acceptance; and 6) extensive post-processing costs in terms of time and money.

LGMD studies have also used timed functional measures, including the 6-min walk test, the timed up and go, the 10-m walk, and the NSAD. These are also widely used within studies for many dystrophies, including the most common variants of DMD, facioscapulohumeral muscular dystrophy (FSHD), and myotonic dystrophy [33–35]. A study on LGMDR2 showed consistently small but clinically meaningful progression over 1 year of follow up, suggesting disease progresses significantly and can be quantified using functional COA [25].

Patient reported outcomes and health impact of disease also become important measures as therapeutic development gets closer to registration [36]. LGMD studies with patient-reported disease impact are currently quite limited. One study used common non-disease specific patient-reported outcomes (PROs) in LGMDs, including the SF-36, the Individual Neuromuscular Quality of Life scale, and screens of fatigue or sleepiness. Not surprisingly, they showed LGMD impacted quality of life mainly in physical areas (weakness, fatigue, and independence), with less consistent findings on emotional or social relationship domains [37].

Early cross-sectional studies have provided preliminary information about the utility of COAs. However, these prior efforts have been conducted by a small number of sites, therefore limiting our ability to define the multi-site reliability and performance of outcome measures. As LGMDs are a rare disease with relatively low population density in any one clinical center, a large cohort prospectively studying the disease longitudinally helps to ensure clinical trial readiness.

### Objectives

The primary goal of this study is to evaluate the utility of a set of outcome measures on a wide range of LGMD phenotypes and ability levels to determine if it would be possible to use similar outcomes between individuals with different phenotypes and to document the natural history of the disease. To this end we will conduct a prospective 12-month study of 188 patients at 12 clinical sites that involves a primary two-armed study. The specific objectives are:

- *Objective 1: Identify, validate, and document the natural history of COAs useful for capturing phenotypic diversity.* We hypothesize that COAs representing the major shoulder and pelvic girdle functional burden can be shared across all LGMDs, and genetic variability can be addressed by adding select mutation relevant COAs to create a LGMD COA toolbox for future trials. Endpoints of 100-m walk, 10-m walk, seated forced vital capacity (FVC), forced expiratory volume in 1 s (FEV1), maximal expiratory pressure (MEP), North Star Assessment for limb girdle-type dystrophies (NSAD), 4 stair climb (4SC), timed up-and-go (TUG), Performance of Upper Limb 2.0 (PUL), 9 hole peg test (9HPT), and hand held dynamometry (HHD) will be used. These measures have previously been evaluated as sensitive to change in other forms of muscular dystrophy. The baseline visit will occur over two days to allow for test–retest reliability of each COA. We will also use biological samples to assess AAV seroreactivity and bank remaining samples for future biomarker discovery. Cross sectional analysis will assess variance between cohorts and within cohorts on select COAs to determine convergent and divergent phenotypes.
- *Objective 2: Determine the sensitivity of the COAs to longitudinal disease progression.* We hypothesize that these COAs will be sensitive to longitudinal disease progression. This idea is supported by numerous studies including efforts by the Clinical Outcome Study for dysferlin (COS) which analyzed many COAs, the University of Iowa which studied longitudinal effects of FKRP mutations, the Jain Clinical Outcomes Study of Dysferlinopathy for NSAD utilization and validation, and natural history multicenter studies on LGMDR1 in France. Data has shown small yet consistent changes in COAs during their 2-year time period, with data being statistically significant as early as 6 months for most mutations but earlier for FKRP (LGMDR9) and CAPN3 (LGMDR1) mutations [38, 39]. We will determine the responsiveness of our COAs at 3, 6, and 12 months. We will use anchoring methods to determine what will be a minimally important clinical difference. We will use factor analysis to identify COAs that capture variation in disease severity across the cohort.
- *Objective 3: Define clinical trial strategies based on baseline and longitudinal phenotypic characteristics.* We will determine which COA are applicable to each subtype of LGMD. We will determine which baseline characteristics, mutation, age, gender, and baseline functional status are most likely to predict progression over 12 months using regression trees to assess subgroups likely to progress.
- *Objective 4: Collect biological samples for biomarker discovery and other future research.* This study prospectively collects blood, urine, and tissue samples that will be stored in a biorepository to provide samples for future LGMD research developments.

## Methods/design

### Study design

**Fig. 1:**
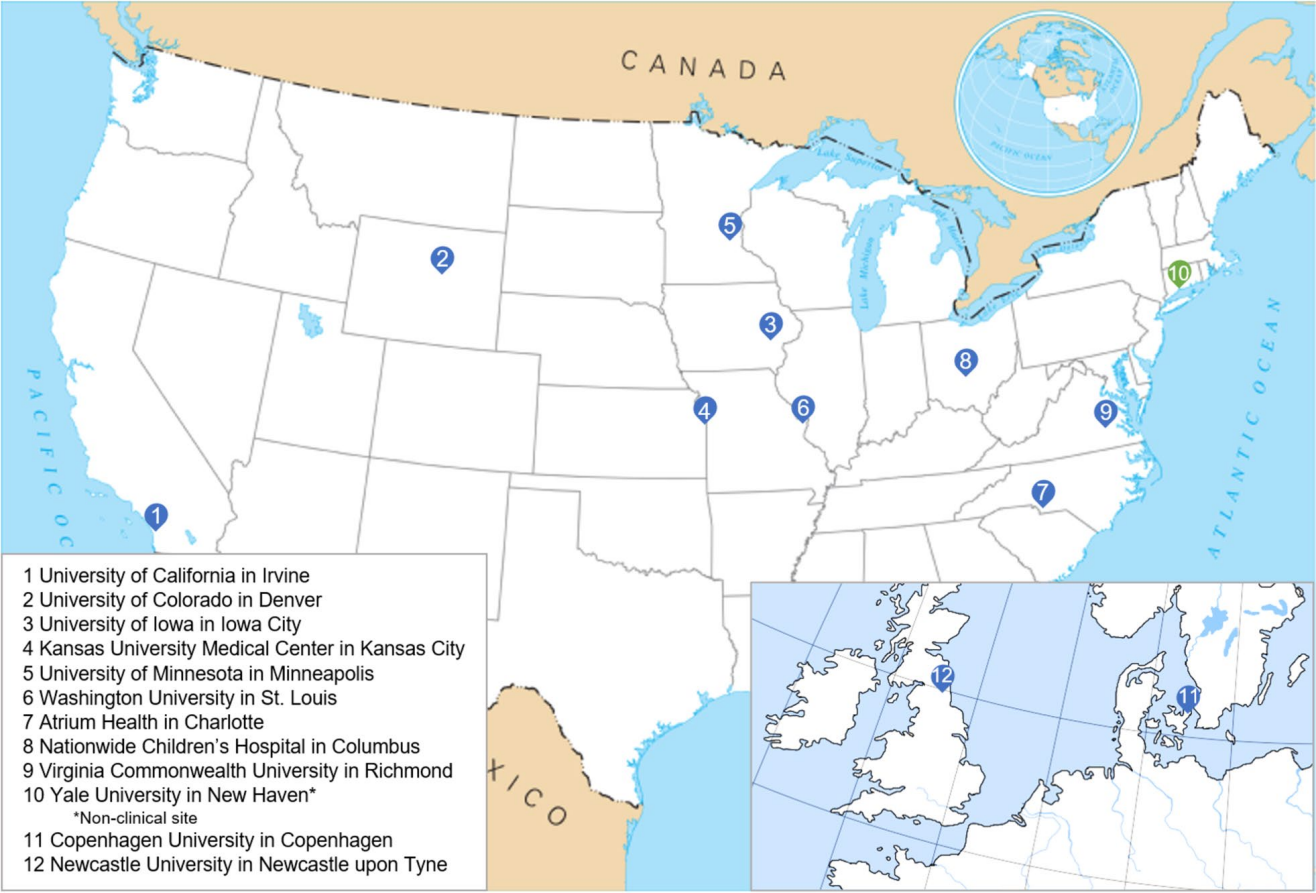
GRASP-LGMD Consortium participating institutions. Adapted from Wikimedia Commons [40, 41]

This is a prospective, multi-center, 12-month study of 188 LGMD patients that is part of the established Genetic Resolution and Assessments Solving Phenotypes in LGMD (GRASP-LGMD) Research Consortium, a network of academic investigators working to improve clinical care and therapeutic development for LGMD (see Fig. 1). The study start date was 6/14/2019 with an estimated completion date of 12/31/2024. Participant enrollment began March 2020 and will complete by August 2024.

### Enrollment

Subject enrollment will include both males and females with the following LGMD variants: LGMD2A/R1, LGMD2B/R2, LGMD2C-E/R3-6, LGMD2L/R12, and LGMD1D/D1. These subtypes were enrolled as they are the most common or the most likely to have near term therapeutic trials. Enrollment will be stratified into individual arms per subtype. Study visits will occur at Baseline Day 1, Baseline Day 2, Month 6, and Month 12 for subjects enrolling with a recessive form (LGMD2/R) and at Baseline Day 1, Baseline Day 2, and Month 12 for dominant forms (LGMD1/D). Of note, SGC mutations will also have a Month 3 visit.

Our approach will utilize an industry-level standard for multisite training to ensure high quality data and results generalizable to subsequent clinical trials. A pre-study multi-site training meeting will convene in Year 1 to review the protocol and train clinical evaluators (CE) and investigators. This meeting will occur at one of the study site locations to ensure access to volunteers with a neuromuscular disorder to establish reliability. Additionally, sites will be contacted at least monthly to review recruitment, enrollment, and study procedures. Rigorous study training will be conducted according to published guidelines including study specific, didactic, and inter-rater reliability testing [42, 43].

Only experienced CEs who have been trained by the study leads will perform the functional assessment. A yearly meeting will occur throughout the conduct of the project to review the protocol and refresh training of COAs. Any CE whose reliability is deemed inadequate will be retrained.

### Subject recruitment and consent

Study participants will be identified through self-selection and direct recruitment options. Participants will be identified from a population of individuals who are followed at the neuromuscular clinics at study sites. The sites were chosen due to wide geographic distribution, experience with LGMD clinical research, prior collaboration work, and access to LGMD patients. Informed consent (and assent for the older cohort) will be obtained by the principal investigator or approved study staff after thorough description of the study. This includes the purpose, procedures, risks, benefits, contact persons, compensation, care for injury, and voluntary participation in the study. An opportunity to ask questions and have their questions answered will be included in the consent process.

### Study population

The subject selection criteria functions to recruit subjects that mirror the types of subjects likely to be selected for LGMD clinical trials. Due to remaining questions about ideal eligibility criteria, the criteria remain broad enough to investigate demographic and genetic associations with disease progression.

### Inclusion and exclusion criteria

See Table 1 for inclusion and exclusion criteria of the study. Individuals who do not initially meet the criteria for participation in this study (screen failure) may be rescreened up to two additional times. The interval between rescreening should be at least 4 weeks after signing the prior consent and involves new consent forms and a new identification number. A participant is not considered enrolled until eligibility is confirmed and study procedures have begun.

**Table 1:** Inclusion and exclusion criteria

Inclusion criteria	Exclusion criteria
Age between 4–65 at enrollment	Any other illness that would interfere with the ability to undergo safe testing or would interfere with interpretation of the results in the opinion of the site investigator
Clinically affected (defined as weakness on bedside evaluation in either a limb-girdle pattern or in a distal extremity)	
Genetically or functionally confirmed mutation in ANO5, CAPN3, DYSF, SGCA-G, or DNAJB6	History of • Bleeding disorder • Platelet count < 50,000 • Current use of an anticoagulant
Willing and able to give informed consent and follow all study procedures and requirements	Positive pregnancy test at any time point during the trial

The age range (4–65 years) is designed to capture the full range of disease severity associated with ambulation status while also reflecting potential clinical trial populations. The younger age limit was chosen to ensure the ability to comprehend the outcome measures proposed. Those with ANO5 (LGMDR12) mutations may not lose ambulation or even be clinically affected until late in life, necessitating an age range of up to 65. The upper age limit also reflects the potential exclusion criteria of future therapeutic trials.

The inclusion criteria do not specify ambulation status. Participants who cannot complete the 10 m walk test as well as participants who are at risk for losing ambulation (10-m walk > 12.9 s) will be allowed to enroll but cannot comprise more than 20% of the overall participant enrollment per arm. This cohort will be referred to as “non-ambulant” for the purpose of this paper. This will improve our understanding of the phenotypic spectrum and allow for evaluation of clinical outcome assessments in patients with advanced disease progression.

### Assessments

Enrolled subjects will be seen at participating sites for baseline evaluation over a two-day period. Follow-up evaluations will occur at months 3, 6, and 12 depending on the specific mutation being studied. Evaluations will include the schedule of activities present in Table 2.

**Table 2:** Schedule of events

Visits	Baseline day 1	Baseline day 2	Month 3^a^ ± 21 days	Month 6^b^ ± 21 days	Month 12 ± 21 days
Informed consent	X				
Demographic information	X				
Medical and surgical history	X				
Inclusion exclusion	X				
Vital signs	X	X	X	X	X
Physical exam	X^c^	X^c^	X	X	X
Pregnancy test^d^	X		X	X	X
10 Meter walk	X				
100 m timed	X	X	X	X	X
NSAD	X	X	X	X	X
4 stair climb	X	X	X	X	X
Timed Up and Go (TUG)	X	X	X	X	X
Handheld dynamometry	X	X	X	X	X
Performance Upper Limb PUL 2.0	X	X	X	X	X
9 hole peg test	X	X	X	X	X
Spirometry	X	X	X	X	X
Concomitant meds	X	X	X	X	X
Domain delta questionnaire		X	X	X	X
Patient reported outcome measures	X^c^	X^c^	X	X	X
Urine biomarkers		X		X	X
Blood biomarkers		X	X	X	X
Prior muscle biopsy acquired (if performed)	X				
Review adverse events		X	X	X	X

^a^For individuals with SGCA-G pathogenic variants

^b^n/a to DNAJB6/ LGMDD1cohort

^c^Assessment can occur on baseline day 1 or baseline day 2

^d^Pregnancy test for women of childbearing potential

### Safety assessments

Due to the non-interventional nature of this study, safety assessments will be conducted in relation to procedures and tests. This includes monitoring of adverse events, concomitant medications, vital signs, and exam findings. For this study, an adverse event is any untoward medical occurrence in a subject during participation in the clinical study visits, tests, and procedures.

In addition to demographic information, a complete medical and surgical history will be obtained to capture other medical comorbidities. Medical history will be updated at each visit to collect ongoing and new events that occur outside of study participation. Clinically relevant prior treatments received by the patient include 1) previous LGMD treatments (including SoC) with patient’s response and reason for changing treatment/dose in the last 12 months and 2) non-LGMD treatment in the last 6 months. This information will be recorded in the electronic case report forms (eCRFs) and will include start and stop dates (as applicable or will state if continuing as concomitant medication). A brief physical exam will also be conducted at each visit to establish baseline values. Vital signs will be also recorded at each visit, including blood pressure (systolic and diastolic), respiratory rate, pulse rate, body temperature, body weight, and standing height or ulnar length. Urine pregnancy testing will be conducted only for females of childbearing potential.

### Efficacy assessments

#### 10-meter walk/run (10 MWR, baseline day 1 only)

The test will be conducted in a quiet hallway and the participants will be instructed to traverse the 10-m course as quickly as possible without using assistive devices. This method has been previously demonstrated to be a good assessment for stability and burst performance capabilities [27, 28].

Prior studies in LGMD have shown that participants could be divided based on their performance on the 10-m walk/run (10 MWR). In the Jain sponsored study of dysferlin performance over time, the 10 MW can be roughly split at 12 s at baseline: those performing the test in under 12 s had more consistent progression rates during follow up, whereas participants with > 12 s at baseline had very high variability in performance or lost the ability to do the task [25].

For this reason, the study divides its participants into two cohorts based on performance under or above 12.9 s. The use of the 12.9 s cut-off allows us to maximize our strategy for validation of relevant COAs to focus testing on muscle groups that can be measured across the study (i.e. shoulder and arm function for those in whom standing and walking activities are not safe}. This will more closely match an industry approach to inclusion in studies, allowing us to maximize our numbers for each outcome while reducing the burden of testing.

### 100-meter timed test (100 m)

The participant will be asked to complete 2 laps around 2 cones set 25 m apart as quickly and safely as possible, running if able. The 100 m is a valid, reliable, and sensitive test to change over time in individuals with Duchenne muscular dystrophy [44]. The 100-m walk has the benefit of reducing the ceiling effect of assessments, as muscular dystrophy patients are performing well below expectations for their age across the lifespan.

### North star assessment for limb girdle-type dystrophies (NSAD)

The NSAD is a functional scale specifically designed to measure motor performance regarding gait and mobility in individuals with LGMD. Despite differences in age of onset, rate of progress, and muscle weakness patterns in individuals with LGMD, the NSAD has been found to be a reliable and valid clinical tool worth inclusion in both clinical practice and research for assessment of muscle weakness via motor performance [45]. It consists of 29 items that are considered clinically relevant from the adapted North Star Ambulatory Assessment and the Motor Function Measure 20 with a maximum score of 54. A higher score indicates higher functional abilities. Previous studies have shown that the NSAD score decreases with age in patients with LGMD [46].

### Performance of upper limb 2.0 (PUL)

The PUL is a tool designed for assessing shoulder/arm function in persons with neuromuscular disorders [47]. It was developed as a conceptual framework reflecting the progression of weakness and natural history of functional decline in DMD. There are 22 scored items. A score of 42 indicates the highest level of independent function and 0 the lowest. Previous studies have shown that the PUL score decreases with age in patients with LGMD [48]. The PUL has been shown to still have room to decline when other testing, such as the NSAD, has reached a floor.

### Timed up-and-go (TUG)

The TUG records the fastest time a patient can stand from chair, walk 3 m, and return to sitting as quickly as possible without use of assistive devices. This test has been shown to effectively evaluate pelvic girdle strength and steadiness of gait to help determine overall ambulatory status in DMD [49].

### 4 stair climb

This test for ambulatory status involves participants ascending 4 steps as quickly and as safely as possible. Participants are permitted to use handrails if needed. This measurement has been used in previous studies specifically for pelvic girdle assessment [27, 31].

### 9 hole peg test

The 9 Hole Peg Test (9HPT) is a quantitative measure of distal upper extremity function. It measures the time required for patients to place 9 pegs in 9 holes on the board and then remove them as quickly as possible. This test is a standard measure of upper extremity function [50].

### Handheld dynamometry (HHD) and pinch grip

Handheld dynamometry using the MicroFET2 myometer will be utilized to capture isometric strength in target muscle groups. Maximum strength will be reported for each muscle group providing a continuous scale variable for analysis.

### Measures of pulmonary function

Spirometry will be performed in a sitting position using standardized equipment. Forced vital capacity (FVC), forced expiratory volume in 1 s (FEV1), and maximal expiratory pressure (MEP) will be assessed. Use of nocturnal or daytime positive pressure ventilation (PPV) (e.g., BiPAP or CPAP) will also be recorded.

### Patient reported outcome measures

Patient-reported disease impact is important for supporting drug registration, especially in slowly progressive diseases like LGMD. Several standard and novel PROs will be included in this study. The best will be selected for each LGMD subtype based on responsiveness to disease progression or cross-sectional associations to severity. The ACTIVLIM, a patient-reported measure of activity limitations, is used to assess individuals with upper and/or lower limb impairments. This measures the ability to perform daily activities. The Disabilities of the Arm, Shoulder, and Hand Questionnaire (DASH) will be implemented to reliably measure levels of disability in an individual’s upper extremity. The Patient Reported Outcomes Measurement Information System 57 (PROMIS-57), a set of patient-reported measures developed by the National Institute of Health, evaluates physical, mental, and social health. This will assist in a more well-rounded understanding of subjects’ conditions. The Limb Girdle Muscular Dystrophy Health Inventory (LGMDHI) is a disease specific questionnaire being developed and validated in this study. The Domain Delta Questionnaire will also be used as a patient-reported measure of their health over 12 months. This will provide a benchmark for the smallest detectable clinically meaningful change on PROs and functional outcome measures. Items included within the surveys will be determined based on appropriate age and abilities.

### Biomarker assessments

A blood sample will be collected at the baseline visit to obtain DNA samples for biomarker discovery. Serum and urine will be collected at each in-person visit for other exploratory biomarkers. Individuals with a prior muscle biopsy will be asked to provide this sample for future research and discovery into biomarkers. These samples will be stored in the biorepository and provide the foundation for future pilot projects and serve as a resource to the greater LGMD community.

### Statistical considerations

#### Reliability and validity of the COAs for each site and overall

Reliability measures the consistency between COAs at two separate occasions and will be measured using methods described in Fleiss and those in Altman and Bland [27, 51]. These analyses include the mean absolute difference, technical error, and intra-class correlation coefficients to account for the degree of partitioned variance between the subjects and the methods [52]. The mean absolute difference is the sum of the absolute paired differences divided by the total number of subjects. The technical error is the square root of the sum of squares of the differences between paired measurements. Intra-class correlation coefficients (CR%) are obtained from a nested analysis of variance of a random effects model in which the repeated measurements are nested within subjects. The CR% is the percentage of the total variation, sum of the between- and within-subject variance, explained by the between-subject variance. The larger the between-subject variance, the smaller the proportion of the total variation contributed by the within-subject variation (paired measurements from different days) and thus, the better consistency between the 2 days. Ninety-five percent lower confidence bounds will be computed for these quantities.

#### Analysis of baseline measures

The cross-sectional data obtained in 188 LGMD patients at baseline will be used to describe the sample and examine the relationships between the COAs and genetic mutation. A factor analysis will be performed to examine the structure of the different COAs, proximal hip girdle or distal, and to determine whether the different components group together in a logical manner; Cronbach’s α will be used to assess the internal consistency of the scale. We will also compute a 95% confidence interval (CI) for the statistics. We will compare baseline differences among groups using analyses of covariance (ANCOVA). The variables to be compared are those with potential effects on COAs. If baseline differences in these variables exist, adjustments will be made by incorporating these variables as covariates in the analysis.

Relationships between the COAs and other variables such as age, gender, age at symptom onset, years since symptom onset, and years since diagnosis will be similarly examined, but these analyses will be exploratory in nature since these associations are not necessarily expected to be strong. For example, it may be that subjects who have a longer duration of symptoms are not necessarily more severely impacted by the disease because, despite their duration of symptoms, they have retained a sufficiently high level of function (e.g., independent ambulation) to qualify for the study. Those who have had symptoms for a longer period of time may also be more likely to participate if their symptoms are milder. The eligibility criteria and bias in identifying subjects may thus have an impact on these relationships.

The assumptions underlying all statistical models will be thoroughly checked using appropriate graphical and numerical methods. In the face of nonlinearity or non-normality, appropriate remedial measures (e.g., variable transformation) will be attempted. If outliers or influential cases are detected, the accuracy of the data or special circumstances surrounding the cases (e.g., the use of aids such as a cane or leg bracing to improve function) will be investigated. If no errors are found, the analyses may be repeated after removing these cases to evaluate their impact on the results; however, the final analyses will include these data points.

#### Analytical plans to manage attrition

For participants who leave the study, their reasoning will be explored, documented, and evaluated by the investigators. We will compare data for those who drop out with those who complete the study. If the reasons or mechanisms for drop out pertain to the study outcomes, a separate analysis will be performed for subjects who complete the study and for subjects who drop out.

#### Responsiveness to change

Changes over time from baseline to 12 months of the outcome COA measures and evaluation conditions (i.e., remote assessment, onsite assessment) will be analyzed using a longitudinal model approach. This longitudinal model approach allows the examination of patterns of change in COA; to detect how these changes relate to covariates such as age, sex, race/ethnicity; and how continuous covariates such as BMI affect these changes. The model imputes missing values and includes measurements made at various time intervals as well as time-varying covariates. The model can be expressed as: ${y}_{ijk}=\mu +{\alpha }_{i}+{\beta }_{j}+{\gamma }_{ik}+{\delta }_{ij}{x}_{ijk}+{\varepsilon }_{ijk}$, where ${y}_{ijk}$ is the dependent variable, i.e., serial data of COA; $i$ denotes the variables, i.e., each COA; $j$ denotes the subject; and $k$ denotes the time, baseline and 12 months; ${x}_{ijk}$ represents the covariates, and ${\varepsilon }_{ijk}$ is an error term. ${\gamma }_{ik}$ reflects the value of the dependent variable when a significant difference occurs between the subjects with, for example, male or female gender. Using the model, we will estimate the changes over time in each COA and the timing when the effects of, for example, gender significantly affects the COAs. If the variables are not normally distributed, generalized estimation equations will be employed [38].

#### Responsiveness to change over time and minimal clinically important changes (MCICs)

In the absence of an intervention known to have a beneficial effect in LGMD, responsiveness of the outcome measures to change over 12 months will be assessed. This is reasonable under the assumption that measurable progression occurs in LGMD over a period of 1 year. Paired t-tests will be used to assess the null hypothesis of zero mean change at 12 months for each measure. Various statistics can be used for quantifying responsiveness, and the effect size and standardized response mean have been recommended for this purpose. The effect size is defined as the mean change divided by the standard deviation of the baseline value. The standardized response mean is defined as the mean change divided by the standard deviation of the changes from baseline. The bootstrap resampling technique will be used to perform formal statistical comparisons among the different outcome measures in terms of these two measures of responsiveness. With this technique, one takes a random sample with replacement of the participants, including their outcomes (bootstrap sample). With this bootstrap sample, the difference in, say, effect size between any two outcome measures is recorded. The process is then repeated a large number of times, with the difference in effect size between the COAs recorded each time; the histogram of these differences in effect size over all bootstrap samples approximates the sampling distribution of the differences in effect size between the COAs. The mean of this approximate sampling distribution (bootstrap distribution) and a 95% confidence interval (obtained using the 2.5th and 97.5th percentiles of the bootstrap distribution) summarizes the results. If the confidence interval does not contain the value of zero, the conclusion is that there is a significant difference in average effect size between the COAs.

Anchor-based and distribution-based methods will be used to determine the MCICs on the COAs and the PROs. Mean responses on the COAs will be described for each of the categories of the domain delta questionnaire (e.g., unchanged, a little better, a lot better, etc.). Receiver operating characteristic (ROC) curve methods will be used to select a cut-off for the 12-month changes in the COAs that is best at minimizing misclassification error, i.e., best distinguishes those who indicate that they are at least “a little better” on the domain-delta questionnaire and those who indicate otherwise. The 12-month changes in the COAs that correspond to effect sizes ranging from 0.30 to 0.50 standard deviation units will also be described and compared to the MCIC identified by ROC curve methods. Anchor-based and distribution-based methods are known to have strengths and limitations, and examination of the results derived by both methods will be useful in reaching consensus on recommendations in this regard for future trials in LGMD.

Although there is significant variability in disease progression in LGMD, little is known about factors that might account for some of this variability. Identification of such factors may help in the design of future clinical trials. For example, important predictors of outcome could be used as stratification variables in the randomization plan and as covariates in the statistical analysis of the primary outcome variable, which would improve the precision of the estimated treatment effect. The baseline variables of primary interest include the COAs, age, gender, age at symptom onset, years since symptom onset, and mutation class. A multiple regression model will be constructed, and competing models will be evaluated using a best-subsets regression technique, in conjunction with Akaike information criterion (AIC) and the Bayesian information criterion (BIC). This information will be combined with clinical judgment to arrive at a final model. Appropriate model checking will be performed, as described above. Regression trees use recursive partitioning to partition the sample into different subsets that have various levels of mean change in the COAs. A strength of these methods is that a cross validation procedure is available for checking the final tree, which can be pruned back to avoid overfitting.

#### Sample size

In this study, the primary clinical outcome assessment (COA) for power and sample size calculations is the NSAD composite. We have combined the LGMDs for power and sample size calculations. We anticipate, based on numerous clinical similarities, that longitudinal analyses will benefit from the increased power from the combined cohort. If the cohorts demonstrate variance on a given analysis, the combined data will not be pooled, and each cohort will be treated separately. Assuming a mean (sd) NSAD of 27 (4) at baseline, a clinically significant 10% change in the NSAD at 12 months would translate to 1.38 change in the mean value. Due to the non-normally distributed values of the NSAD, we propose using a Wilcoxon signed-rank test to look at the paired differences in subjects from baseline to the 12-month assessment. Assuming a similar standard deviation, we have a standardized effect size, *dz*, of 0.345. In addition, the study is powered to discern different rates of progression. Using an alpha 0.05 significance level, a power of 95%, and a two tailed test, we calculate that we will need a sample of *n* = 150 to discriminate on the rates of progression. To accommodate up to 20% attrition in subjects over the course of the study, we propose recruiting a total sample of *n* = 188 subjects. R version 4.2.3 was used to calculate the sample size.

## Discussion

To the best of our knowledge, this will be the largest COA development study performed in LGMD at its completion. The large sample size in a study with common COAs and industry standard training will permit important analytical approaches to overcome phenotype variability and validate COAs for future clinical trials in LGMD. In addition to identifying where LGD subtypes converge and can use common COAs, this study will develop strategies to add COAs for where LGMD phenotypes diverge, and determine the baseline factors (i.e., mutation, age, baseline functional status) which might predict progression. This project will hasten therapeutic development via more effective clinical trials by providing information for valid and reliable outcome measures, sample size calculations, and eligibility criteria.

There are limitations to this study. The sample size selected for this LGMD study may not be truly representative of the LGMD population as a whole. For example, muscular dystrophy studies have not had sufficient minority representation in the past. We are actively working to diversify the subject population in this study through more widespread recruitment. Additionally, reliability is a concern due to this being a multi-site study composed of various clinical evaluators and investigators. Aiming to mitigate this concern, this study utilizes industry standards for multi-site studies that strives to standardize data collection. Future clinical trials for LGMD drug development will most likely have similar challenges. It is possible the timeframe may be too short to detect change in some of the slowly progressive LGMDs. If this is the case, we will extend the study visits beyond 12 months.

This study also has many distinguishing characteristics that should be mentioned. This study is conducted by the GRASP-LGMD Research Consortium. This network, through leadership by academic investigators, collaborates with industry, academic institutions, and LGMD patients to improve clinical care and therapeutic development of LGMD patients. Information obtained from this study will be made available for any investigator or company pursuing treatments for LGMD. The creation of a biorepository during this project, despite not being necessary for this study, will provide unique samples for future investigations. This project also works to improve relationships between the network and patients and families via increased patient engagement. Patients provide invaluable information in regard to what is clinically meaningful to them. Their input also helps address issues concerning subject recruitment and retention.

Overall, our aim is to use the strengths afforded by the expansive GRASP-LGMD Research Consortium network along with patient engagement to advance drug development and, therefore, improve patient outcomes. More specifically, this study will help address challenges in LGMD clinical trial preparedness by determining the presence and strength of various COAs among the four most common types of LGMDs.

## Data Availability

Not applicable.

## Abbreviations

10: MWR 10-m walk/run
100m: 100-Meter timed test
4SC: 4 Stair climb
9HPT: 9-Hole peg test
AIC: Akaike information criterion
ANCOVA: Analyses of covariance
BIC: Bayesian information criterion
BiPAP: Bilevel positive airway pressure
CE: Clinical evaluators
CI: Confidence interval
COA: Clinical outcome assessments
COS: Clinical outcome study
CPAP: Continuous positive airway pressure
CR%: Intra-class correlation coefficients
DASH: Disabilities of the Arm, Shoulder, and Hand Questionnaire
DMD: Duchenne Muscular Dystrophy
eCRFs: Electronic case report forms
FEV1: Forced expiratory volume in 1 s
FSHD: Facioscapulohumeral muscular dystrophy
FVC: Forced vital capacity
GRASP-LGMD: Genetic Resolution and Assessments Solving Phenotypes in Limb Girdle Muscular Dystrophy
HHD: Handheld dynamometry
LGMD: Limb Girdle Muscular Dystrophies
LGMDHI: Limb Girdle Muscular Dystrophy Health Inventory
MCIC: Minimal clinically important changes
MEP: Maximal expiratory pressure
MRI: Magnetic resonance imaging
NSAD: North Star Assessment for Limb Girdle-Type Dystrophies
PPV: Positive pressure ventilation
PRO: Patient reported outcomes
PROMIS-57: Patient Reported Outcomes Measurement Information System
PUL: Performance of upper limb 2.0
ROC: Receiver operating characteristic
SMA: Spinal Muscular Atrophy
TUG: Timed up-and-go
